# Speech Outcomes in 5-Year-Old Korean Children with Bilateral Cleft Lip and Palate

**DOI:** 10.1055/a-2175-1893

**Published:** 2024-02-07

**Authors:** Kyung S. Koh, Seungeun Jung, Bo Ra Park, Tae-Suk Oh, Young Chul Kim, Seunghee Ha

**Affiliations:** 1 Department of Plastic and Reconstructive Surgery, University of Ulsan, College of Medicine, Asan Medical Center, Seoul, Korea; 2 Division of Speech Pathology and Audiology, Audiology and Speech Pathology Research Institute, Hallym University, Chuncheon, Korea; 3 Department of Rehabilitation Medicine, University of Ulsan, College of Medicine, Asan Medical Center, Seoul, Korea

**Keywords:** bilateral cleft lip and palate, speech outcomes, 5-year-old, perceptual ratings, cleft audit protocol

## Abstract

**Background** Among the cleft types, bilateral cleft lip and palate (BCLP) generally requires multiple surgical procedures and extended speech therapy to achieve normal speech development. This study aimed to describe speech outcomes in 5-year-old Korean children with BCLP and examine whether normal speech could be achieved before starting school.

**Methods** The retrospective study analyzed 52 children with complete BCLP who underwent primary palatal surgery at a tertiary medical center. Three speech-language pathologists made perceptual judgments on recordings from a speech follow-up assessment of 5-year-old children. They assessed the children's speech in terms of articulation, speech intelligibility, resonance, and voice using the Cleft Audit Protocol for Speech-Augmented-Korean Modification.

**Results** The results indicated that at the age of five, 65 to 70% of children with BCLP presented articulation and resonance within normal or acceptable ranges. Further, seven children with BCLP (13.5%) needed both additional speech therapy and palatal surgery for persistent velopharyngeal insufficiency and speech problems even at the age of five.

**Conclusion** This study confirmed that routine follow-up speech assessments are essential as a substantial number of children with BCLP require secondary surgical procedures and extended speech therapy to achieve normal speech development.

## Introduction

The cleft type has been reported to be a significant influential factor in the clinical outcomes for individuals with cleft lip and/or palate. 1 2 3 Bilateral cleft lip and palate (BCLP), which results in the most severely affected speech mechanism among the cleft types, generally results in less favorable outcomes and requires multiple surgical procedures and extended speech therapy to achieve normal speech development. According to an annual report of the Cleft Registry and Audit Network (2020) in the United Kingdom, 4 25.5% of those with BCLP underwent secondary palatal surgery and fistula repair before 5 years of age. Only 35.1% of children with BCLP were reported to have normal speech compared with 71.9% of the children with unilateral cleft palate. 4 Baillie and Sell also reported that only 39% of the BCLP group had normal articulation at 5 years of age, 5 in contrast to 69 and 85% of the unilateral cleft lip and palate and solitary cleft palate groups, respectively.

Although a few studies have reported the speech outcomes of individuals with BCLP compared with those of unilateral cleft lip and palate or cleft palate only, 2 5 6 7 8 the number of children with BCLP included in these studies is relatively less. In particular, even fewer studies have examined outcomes of children with BCLP, as a separate group, using not only an examination of hypernasality or resonance issues owing to velopharyngeal insufficiency (VPI), but also a detailed evaluation of the consonant sound system. Recently, the number of clinical audits related to cleft lip and/or palate has increased to allow quality improvement, which will help improve outcomes in children with cleft palate. Such efforts have been made mainly in the context of the Cleft Registry and Audit Network of the United Kingdom and the Scandcleft Project in the European countries. The surgical protocols vary according to cleft type between surgeons, from unit to unit, and across countries. 9 10 11 Therefore, it is important to audit clinical outcomes of children with cleft lip and/or palate from various institutions and countries and meticulously monitor their speech development.

Five years of age, which is around the time when children enter elementary school, is a critical period from the perspective of a child's overall development. At this age, children are ready to learn new academic skills and engage in school life to reach developmental milestones in terms of physical, cognitive, language, and social skills. In particular, children acquire their speech sound system and show stable speech production at 5 years of age, which play a role in establishing the foundation of literacy and moving forward to the next developmental stage in terms of academic, social, and emotional aspects. Likewise, most Korean-acquiring children typically pronounce all Korean consonants and vowels accurately by the age of 5 years, except for some children who show residual sibilant distortion. Given the significance of this age in child development, many studies focus on the speech outcomes of 5-year-old children with cleft palate. For example, the Cleft Registry and Audit Network's annual report audit outcomes, including children's growth, dental health, facial growth, speech, and psychology, among children of 5 years of age ( *https://www.crane-database.org.uk/* ). The Scandcleft studies have also mainly reported speech outcomes in 5-year-old children with unilateral cleft lip and palate. 12 13 14

A listener perceptual evaluation is the standard clinical assessment procedure for reporting speech outcomes of children with cleft palate. 15 16 Many centers in various countries currently use the Cleft Audit Protocol for Speech-Augmented (CAPS-A) or the modified version of the CAPS-A tool to report speech outcomes of children with cleft palate. 17 18 19 The Korean modified version of CAPS-A was also developed to assess the speech of Korean children born with cleft palate. 18 The assessment tools rely heavily on the listener perceptual ratings of various speech parameters; therefore, the reliability of the judgment of perceptual ratings is essential to improve the value of perceptual studies on cleft palate speech. Brunnegård and Lohmander (2007) identified several criteria for listener perceptual ratings. 16 They highlighted the importance of reporting intra- and interrater reliability from multiple raters and recordings that are randomized and blindly assessed. They also suggested that researchers should use a narrow age span for the studied group and report the inclusion or exclusion of speakers with additional anomalies or cognitive delays.

The current study aimed to investigate speech outcomes in a group of 5-year-old Korean-acquiring children with BCLP and examine whether they achieve normal speech before starting school. This study also allowed us to audit clinical care outcomes in Korean-speaking children with BCLP using the Cleft Audit Protocol for Speech-Augmented-Korean Modification (CAPS-A-KM), 18 which makes it possible to compare clinical outcomes between institutions with different cultural and linguistic backgrounds while simultaneously considering the potential effects of a language-specific phonological system on Korean children's speech developments.

## Methods

### Participants

Approval for the study was obtained from the Institutional Review Board of this medical center (approval number: 2020-0913).

This study involved a retrospective analysis of a consecutive series of patients who underwent primary palatoplasty in the Cleft Palate-Craniofacial Clinic in our center between 2010 and 2017. Fifty-two children with complete BCLP were eligible for inclusion in the study. Children met the inclusion criteria if they had a diagnosis of complete BCLP, had complete speech assessment recording data at the age of 5 years, and had no known medical diagnoses, including sensorineural hearing loss, intellectual impairment, or syndromes. The children with BCLP included 44 boys and 8 girls. Cheiloplasty was performed at a mean age of 88.9 days (range: 69–126 days) for all children in the study. They underwent primary palatoplasty at a mean age of 11.94 months (range: 10–17 months). The surgeries were performed by two surgeons in a tertiary medical center, where approximately 16.2% of all primary palatoplasty operations in South Korea between 2010 and 2017 were performed ( *https://www.opendata.hira.or.kr* ). Primarily, one surgeon (K.S.K.) performed the primary palatoplasty in all children with BCLP except for three children, whose operations were completed by the other surgeon (T-S.O.). All participants underwent three-flap ( *n* = 38, 73.1%), modified two-flap ( *n* = 11, 21.2%), Furlow double opposing Z plasty ( *n* = 1, 1.9%), or two-stage palatoplasty ( *n* = 1, 3.8%; soft palate closure at 9–12 months of age and hard palate closure around 18 months of age). Staged palatoplasty was performed due to limited access to the hard palate caused by restricted mouth opening.

Secondary palatal surgery was indicated when patients showed mild-to-moderate or higher degree of hypernasality due to VPI. The need for secondary palatoplasty between 3 and 5 years of age was evaluated based on comprehensive results of perceptual speech assessments, nasometer, and if possible, nasoendoscopic evaluation. If patients showed compensatory articulation problems as well as mild-to-moderate hypernasality, they were asked to receive speech therapy for 6 to 12 months. Secondary palatal surgery was considered the next step if speech outcomes were limited and the degree of hypernasality was not improved.

### Data Collection

All children in the study attended routine follow-up speech assessments at 5 years of age. Either of the two speech pathologists, who were working at the clinic at this time, conducted and recorded the follow-up speech assessments. The speech audio recordings consisted of standard speech samples, including word lists from the standardized articulation test, Urimal Test of Articulation and Phonation, 20 counting from 1 to 10, and repetition of sentences loading mainly high, low, or mixed vowels.

All demographic and medical information, including data on lip and palate repairs, postoperative complications (e.g., oronasal fistula, VPI), and speech therapy, was obtained from patients' electronic medical records.

### Data Analysis

#### Perceptual Assessments

The CAPS-A-KM was used for the analysis of the speech samples. 18 Based on the CAPS-A, 21 the CAPS-A-KM consists of ordinal scales for rating features of articulation, speech intelligibility, resonance, and voice. Articulation errors were categorized into developmental errors and cleft speech characteristics (CSCs; compensatory and obligatory misarticulations). The severity of the error was rated using a 3-point scale according to the number of consonants (0 = absent; 1 = two or fewer different consonants; 2 = three or more different consonants). The consonant error types of the CSCs included mid-dorsum palatalization, backing to a velar or uvular place of articulation, pharyngeal articulation, glottal articulation, posterior nasal nasalization, nasal fricatives, and double articulation. In addition, obligatory or passive misarticulations involved the weakened production of pressure consonants, nasalization of plosives, and the gliding of fricatives or affricates. Speech intelligibility was rated using a 7-point scale (0 = always intelligible; 1 = intelligible but delayed speech; 2 = 75% of speech is intelligible; 3 = 50–70% intelligible; 4 = 30–50% intelligible; 5 = 10–30% intelligible; 6 = always unintelligible). The resonance section included hypernasality, hyponasality, cul-de-sac resonance, and audible nasal emission. Hypernasality was rated using a 7-point scale (0 = normal; 1 = minimal; 2 = mild; 3 = mild-to-moderate; 4 = moderate; 5 = moderate-to-severe; 6 = severe). The two-scale values “normal” and “minimal” were pooled into a single scale value of normal speech when reporting the results. Children showing hypernasality greater than a mild-to-moderate degree were usually referred for further clinical management in the clinic. Hyponasality, cul-de-sac resonance, and audible nasal emission were rated on a 2-point scale (0 = no occurrence; 1 = occurrence). Voice was rated on a 2-point scale (0 = normal; 1 = abnormal) in terms of loudness, pitch, and quality. Finally, the need for further surgical repair or speech therapy was noted.

Perceptual analysis was performed using the speech audio recordings made from the follow-up speech assessment at 5 years of age. The recordings were converted into mp3 files, and any personally identifiable information of the children as well as the date of recording was removed. The recordings were randomized in order and uploaded into encrypted cloud storage. Approximately 13.5% (seven children) of the recordings were duplicated for intrarater reliability, and the recordings of six 5-year-old children with other medical issues (e.g., syndrome and neuromuscular disorders) were also included in the rating speech samples.

#### Raters

Three speech-language pathologists who conducted the annual follow-up speech assessments performed the analysis. All three individuals were affiliated with the cleft palate team of this medical center and had been involved in several research projects. They had a minimum of 7 years of experience with the perceptual speech assessment in children with cleft palate. They had also completed several training sessions before performing the perceptual analysis for this study. They had practiced with the CAPS-A-KM tool using recordings from 10 children with cleft palate, who were not included in this study, and discussed the rating results. After two training sessions, the three raters performed the perceptual analysis of the recordings separately and independently. They subsequently gathered to review the rating results and, in cases of disagreement, they listened to the recording together and agreed upon a consensus score. Interrater and intrarater reliability were investigated using the original ratings from each rater, but the consensus judgment was used to report speech results for this study.

### Reliability

The duplicated recordings of seven children with BCLP were randomly selected from the entire dataset for measuring interrater and intrarater reliability. Intraclass correlation coefficients (ICCs) which were generally reported in many of the previous speech studies 17 21 22 were calculated for interrater and intrarater reliability. The study utilized a two-way mixed model to assess interrater reliability and a one-way random-effects model to evaluate intrarater reliability. The results showed remarkable intrarater reliability among the three raters, with average measures ICCs of 0.94, 0.97, and 0.98, for each parameter on the CAPS-A-KM. Overall, the findings suggest that the raters demonstrated excellent intrarater reliability in this study. Table 1 shows the interrater reliability results for each parameter on the CAPS-A-KM. The average measures ICC values of the interrater reliability generally indicated moderate-to-excellent agreement, except for some parameters such as certain compensatory articulations (i.e., pharyngeal articulation, nasal fricatives). The occurrences of such parameters were low or nonexistent, and the distributions were skewed, which may result in low values. 16

**Table 1:** Interrater reliability results

Parameters	ICC	Interpretation
Developmental errors	0.69	Good
CSCs (average)	0.47	Moderate
Palatalization	0.72	Good
Backing to velar/uvular	0.76	Good
Pharyngeal articulation	0.35	Fair
Glottal articulation	0.88	Very good
Posterior nasal fricatives	0.66	Good
Nasal fricatives	0.22	Fair
Coarticulation	0.50	Moderate
Passive CSCs	0.50	Moderate
Speech intelligibility	0.84	Very good
Hypernasality	0.90	Excellent
Audible nasal emission	0.75	Good
Voice	0.58	Moderate

Abbreviations: CSC, cleft speech characteristic; ICC, intraclass correlation coefficient.

## Results

### History of Secondary Surgery and Speech Therapy

Before 5 years of age, 51.9% ( *n* = 27) of children had undergone secondary palatoplasty, and all underwent Furlow double opposing Z plasty at an average age of 55.6 months (range: 41–88 months). The incidence of fistula in children with BCLP in this study was 17.3% ( *n* = 9). Of these, fistula repair was done concurrently with secondary palatoplasty in two patients by elevating the mucoperiosteal flap around the defect. In the remaining patients, the presence of fistula was not related to occurrence of hypernasality.

It was difficult to obtain accurate information about the extent and nature of speech therapy from children's medical records. Most speech therapy took place at local speech clinics. Based on the available data, 76.9% ( *n* = 40) had received or at least been referred for speech therapy while 23.1% ( *n* = 12) had not received speech therapy.

### Speech Outcomes from Perceptual Ratings

The speech results were all based on the consensus judgment of the three raters.

#### Articulation

The results showed that 34.6% ( *n* = 18) of the participants presented with no CSCs. Table 2 presents the number and percentage of children showing one or more consonants affected by each CSC category. The most frequent CSC was palatalization (34.6%; *n* = 18), followed by backing to velar/uvular (25.0%; *n* = 13). Of the nonoral errors, glottal articulation (25.0%; *n* = 13) was the most frequent CSC, followed by pharyngeal articulation (19.2%; *n* = 10) and posterior (active) nasal fricative (13.5%; *n* = 7). Six children with BCLP (11.5%) had coarticulation (double articulation), and one child had nasal fricatives affecting one or two consonants. Some children also had passive CSCs, such as weak oral consonants (9.6%; *n* = 5) and nasalization (7.7%; *n* = 4).

**Table 2:** Speech outcomes of 5-year-old children with bilateral cleft lip and palate by each cleft speech characteristic category

Types	≤Two consonants affected		Three or more consonants affected		Total	
	*n*	Percentage (%)	*n*	Percentage (%)	*n*	Percentage (%)
Palatalization	16	30.8	2	3.8	18	34.6
Backing to velar/uvular	11	21.2	2	3.8	13	25.0
Pharyngeal articulation	9	17.3	1	1.9	10	19.2
Glottal articulation	6	11.5	7	13.5	13	25.0
Posterior nasal fricatives	7	13.5	0	0	7	13.5
Nasal fricatives	1	1.9	0	0	1	1.9
Coarticulation	4	7.7	2	3.8	6	11.5
Weak oral consonants	4	7.7	1	1.9	5	9.6
Nasalization	4	7.7	0	0	4	7.7

#### Speech Intelligibility

Table 3 summarizes the results from the perceptual ratings of speech intelligibility. Thirty-six (69.2%) children presented completely intelligible speech, with one or two points on the interval scaling of speech intelligibility. Seven (13.5%) children's speech was 70% intelligible. Nine (9.6%) children showed speech with 70% or less understood words, and two children's speech was barely intelligible, showing only 10 to 30% of understood words.

**Table 3:** Speech intelligibility and hypernasality of 5-year-old children with bilateral cleft lip and palate

Speech intelligibility ratings	*n*	Percentage (%)	Hypernasality ratings	*n*	Percentage (%)
Completely intelligible	13	25.0	Normal	3	5.8
Mostly intelligible despite some errors	23	44.2	Minimal	17	32.7
70% intelligible	7	13.5	Mild	13	25.0
50 to 70% intelligible	4	7.7	Mild to moderate	8	15.4
30 to 50% intelligible	3	5.8	Moderate	8	15.4
10 to 30% intelligible	2	3.8	Moderate to severe	2	3.8
			Severe	1	1.9

#### Hypernasality

Table 3 also presents the results from perceptual ratings of hypernasality. The perceptual ratings indicate that 63.5% ( *n* = 33) of participants had normal-to-mild evidence of hypernasality. Eight children (15.4%) had mild-to-moderate hypernasality. The results also revealed that 21.2% ( *n* = 11) of participants showed a moderate or higher degree of hypernasality that clearly required clinical management—namely, 15.4% ( *n* = 8) had moderate, 3.8% ( *n* = 2) moderate-to-severe, and 1.9% ( *n* = 1) severe.

#### Other Resonance Issues

Of the 52 participants, 8 children (15.4%) had audible nasal air emission or nasal turbulence occurring on oral consonants. Three children (5.8%) showed cul-de-sac resonance, while none showed hyponasality.

#### Voice

Voice problems were identified in terms of loudness, pitch, and voice quality in the CAPS-A-KM. Ten children (19.2%) presented voice quality problems. No participants showed obvious voice problems related to loudness and pitch.

#### Residual Needs for Speech Therapy or Palatal Surgery

The CAPS-A-KM was used to determine residual needs for speech therapy or palatal surgery based on speech outcomes from perceptual ratings. Table 4 summarizes the judgment results. Based on the results, 48.1% ( *n* = 25) required further speech therapy and 15.4% ( *n* = 8) required additional palatal surgery for residual VPI at the age of 5 years. Comprehensively, 26 children (50%) needed neither additional speech therapy nor palatal surgery, and 7 children (13.5%) needed both additional speech therapy and palatal surgery to achieve normal speech at the age of 5 years.

**Table 4:** Residual needs for speech therapy or palatal surgery based on speech outcomes from perceptual ratings

	Surgical procedures for VPI			Total
		Yes	No	
Speech therapy	Yes	13.5% *N* = 7	34.6% *N* = 18	48.1% *N* = 25
	No	1.9% *N* = 1	50% *N* = 26	21.2% *N* = 27
Total		15.4% *N* = 8	84.6% *N* = 44	100% *N* = 52

Abbreviation: VPI, velopharyngeal insufficiency.

## Discussion

This study involved a clinical audit of Korean-speaking children with BCLP who received primary palatal surgery from primarily one surgeon at a single cleft center. This study focused on speech outcomes of children with BCLP at the age of 5 years, a critical period during which children enter school, from the perspective of the child's general development.

Children with BCLP cleft type are born with the most severely affected speech mechanism among the cleft types. Our results confirmed that a substantial number of children with BCLP usually require secondary surgical procedures and extended speech therapy to achieve normal speech development. 4 6 23 24 This retrospective analysis showed that 51.9% of children had undergone secondary palatoplasty at an average age of 55.6 months, while 17.3% had a fistula at 5 years of age. In addition, 76.9% had received or at least been referred for speech therapy before 5 years of age. This result indicates a relatively higher rate of secondary palatoplasty compared with reports from other countries. 4 It is difficult to make direct comparisons among studies as different communities, institutional policies, and attitudes influence clinical management. Children showing mild-to-moderate hypernasality in the cleft clinic of the study were usually referred for Furlow double opposing Z plasty for secondary palatoplasty which are surgical procedures not often considered in other institutions. Therefore, secondary palatal surgery in the cleft clinic tends to be performed rather radically, which might make the rate of secondary palatoplasty relatively higher.

The speech results from the consensus of perceptual judgment by the three raters showed that approximately 65 to 70% of children with BCLP achieve completely intelligible speech by the age of 5 years. This study examined children's articulation errors closely and showed that 34.6% of children with BCLP did not present cleft-related articulation difficulties. Indeed, they showed normal articulation or merely minor developmental articulation errors. This percentage was almost similar to the results of studies reporting cleft speech audits in the United Kingdom, which reported that only 35 to 39% of the BCLP group had normal articulation by the age of five. 4 5 The results of the current study showed that palatalization and backing to velar/uvular were the most prevalent CSCs at the age of 5 years, which is consistent with the results from speech audits in the United Kingdom. 4 5 Both palatalization and backing to velar/uvular are considered “retracted oral articulation.” 13 14 25 26 This result suggests that children with cleft palate, including BCLP, present persistent articulation place errors related to the preference of the posterior area of the oral mechanism. The literature suggests that these articulatory errors are associated with otitis media with effusion (OME). 5 27 Unfortunately, this study could not systematically investigate the history of OME owing to missing or incomplete medical information. Further investigations should be conducted to address the effect of the history of OME on cleft-related speech in this population.

Similar to the results related to speech intelligibility, the perceptual ratings showed that approximately 64% of participants had normal, minimal, or mild evidence of hypernasality, which involves a normal or acceptable range of speech. On the other hand, this study revealed that approximately 21% of children with BCLP showed a moderate or higher degree of hypernasality, which generally requires surgical procedures. The CAPS-A-KM required the raters to determine residual needs for speech therapy or palatal surgery based on the overall speech outcomes. They consistently judged that approximately 17% of the children required additional palatal surgery for residual velopharyngeal insufficiency by 5 years of age.

Seven children with BCLP (13.5%) needed both additional speech therapy and palatal surgery even at 5 years of age. This figure corresponds to the number of children showing severely affected speech intelligibility and a moderate or higher degree of hypernasality. This result indicates that these children had a sufficiently different speech to provoke comments and required protracted clinical management to achieve normal or at least acceptable speech after 5 years of age, which might have a negative effect on their school life. This also suggests that the period of follow-up speech examinations should be extended. For example, the clinical audit from the Eurocleft project included teenage children with cleft palate and reported that most of the study group produced acceptable and understandable speech at 11 to 14 years of age. 28

This study addressed a significant clinical implication in that it used the Korean-modified version of the cleft audit protocol, the CAPS-A-KM, to report speech outcomes in children with BCLP. The tool is consistent with the CAPS-A tool, which is mostly employed by various centers worldwide, thereby enabling us to compare our results with the clinical outcomes of other studies. In addition, this study met several criteria for listener perceptual ratings to improve the value of perceptual studies of cleft palate speech. 16 This study showed stable intra- and interrater reliability related to cleft palate speech and involved recordings that were randomized and blindly assessed. Ruling out confounding factors, this study focused on clinical outcomes from a homogeneous group, that is, 5-year-old children with BCLP without additional anomalies or cognitive delays who received palatal surgical corrections at a single center.

The limitation of this study should be addressed. Although the number of children with BCLP in this study was a great advantage of the investigation as there was only a single cleft type, this aspect could also be a weakness. Further analysis should be conducted considering the extent of the cleft, surgical procedures, and presence of other significant factors. Continuous research efforts should be made to establish optimal clinical management for children with cleft palate.
